# Non-selective beta-blockers and the incidence of hepatocellular carcinoma in patients with cirrhosis: a meta-analysis

**DOI:** 10.3389/fphar.2023.1216059

**Published:** 2023-07-19

**Authors:** Xinyi He, Zimo Zhao, Xi Jiang, Yan Sun

**Affiliations:** ^1^ Department of Gastroenterology, Shengjing Hospital of China Medical University, Shenyang, Liaoning, China; ^2^ Clinical Department I, China Medical University, Shenyang, Liaoning, China; ^3^ First Clinical Medical College, China Medical University, Shenyang, Liaoning, China; ^4^ Department of Cardiology, Shengjing Hospital of China Medical University, Shenyang, Liaoning, China

**Keywords:** adrenergic beta-antagonists, liver cancer, hepatocellular carcinoma, liver cirrhosis, portal hypertension, meta-analysis

## Abstract

**Background:** Hepatocellular carcinoma (HCC) is a serious complication of cirrhosis. Currently, non-selective beta-blockers (NSBBs) are commonly used to treat portal hypertension in patients with cirrhosis. The latest research shows that NSBBs can induce apoptosis and S-phase arrest in liver cancer cells and inhibit the development of hepatic vascular endothelial cells, which may be effective in preventing HCC in cirrhosis patients.

**Aim:** To determine the relationship between different NSBBs and HCC incidence in patients with cirrhosis.

**Methods:** We searched the Cochrane database, MEDLINE, EMBASE, PubMed, and Web of Science. Cohort studies, case‒control studies, and randomized controlled trials were included if they involved cirrhosis patients who were divided into an experimental group using NSBBs and a control group with any intervention. Based on heterogeneity, we calculated odds ratio (OR) and 95% confidence interval (CI) using random-effect models. We also conducted subgroup analysis to explore the source of heterogeneity. Sensitivity analysis and publication bias detection were performed.

**Results:** A total of 47 studies included 38 reporting HCC incidence, 26 reporting HCC-related mortality, and 39 reporting overall mortality. The HCC incidence between the experimental group and the control group was OR = 0.87 (0.69 and 1.10), *p* = 0.000, and I^2^ = 81.8%. There was no significant association between propranolol (OR = 0.94 and 95%CI 0.62–1.44) or timolol (OR = 1.32 and 95%CI 0.44–3.95) and HCC incidence, while the risk of HCC decreased by 26% and 38% with nadolol (OR = 0.74 and 95%CI 0.64–0.86) and carvedilol (OR = 0.62 and 95%CI 0.52–0.74), respectively.

**Conclusion:** Different types of NSBB have different effects on the incidence of patients with cirrhosis of the liver, where nadolol and carvedilol can reduce the risk. Also, the effect of NSBBs may vary in ethnicity. Propranolol can reduce HCC incidence in Europe and America.

**Systematic Review Registration:** identifier https://CRD42023434175, https://www.crd.york.ac.uk/PROSPERO/.

## Introduction

Primary liver cancer is the sixth most common cancer type in the world, ranking third in global cancer-related deaths. Among them, hepatocellular carcinoma (HCC) accounts for 75–85% of all liver cancer cases (Sung et al., 2021). Cirrhosis of any cause will increase the risk of HCC (Hartke et al., 2017). The internal structure of the liver with cirrhosis is greatly changed and different from that of the normal liver. At this point, fibrosis is a basic feature of cirrhosis, which conceals liver cancer in nodules and makes it difficult for our immune cells to recognize mutated tumor cells. Therefore, cirrhosis can easily develop into liver cancer. Similarly, many drugs have difficulty in penetrating the nodules of cirrhosis. Therefore, targeted drugs for liver cancer generally do not work well. In summary, the diagnosis and prognosis of HCC is complex due to the presence of underlying cirrhosis (Kulik and El-Serag, 2019). Therefore, for patients with cirrhosis, prevention is particularly important.

In the past 40 years, non-selective beta-blockers (NSBBs) have been used in patients with cirrhosis for the prevention of variceal bleeding and management of portal hypertension (Lebrec et al., 1980). NSBBs reduce the portal pressure by reducing the portal blood flow. Beta blockers acting on the cardiovascular system β1 receptor can reduce the heart rate and myocardial contractility, resulting in a decrease in cardiac output and systemic circulation blood volume, thereby reducing the portal pressure. Acting on the β2 receptor, the α receptor antagonistic β2 receptor in the visceral vascular bed is relatively excited, resulting in mesentery and other visceral vascular constriction, thus reducing the portal blood flow. In addition, beta-blockers may also be associated with changes in vasoactive substances (Yoon et al., 2021). More importantly, there are many other benefits of NSBB treatment, such as reducing the risk of bacterial infection by improving immune responses, preventing spontaneous bacterial peritonitis by reducing bacterial translocation (Senzolo et al., 2009), improving the overall rebleeding episodes (Senzolo et al., 2006), and ultimately achieving improved survival (Thalheimer et al., 2008). Recently, some studies have shown that NSBB may also play a role in the prevention of HCC in patients with cirrhosis.

A study in the US demonstrated an association between NSBBs and a lower risk of HCC in cirrhosis (Wijarnpreecha et al., 2021). This may be related to the fact that NSBBs can induce apoptosis and S-phase arrest in human HCC cell lines and reduce invasion and migration in liver cancer cells (Işeri et al., 2014; Wang et al., 2018). A 2019 cohort study showed that high cumulative doses of propranolol reduced the risk of HCC in compensated cirrhosis patients without major complications (Yeh et al., 2019). However, another study showed that the use of propranolol was associated with reduced mortality but not HCC development in patients with cirrhosis and refractory ascites (Chen et al., 2022). A previous meta-analysis stated that NSBBs may prevent HCC in patients with cirrhosis, but they did not find an effect of NSBB on HCC-related mortality and overall mortality in the NSBB intervention *vs.* the control group (Thiele et al., 2015). What role does NSBB play in the progression of HCC in patients with cirrhosis, and what changes does it bring about in cancer? There are still many controversies in current studies. The meta-analysis in 2015 was limited by a small number of patients and events due to the majority of eligible trials not registering HCC incidence or HCC mortality, and it only performed an analysis of RCTs before 2015. Therefore, we updated and added to this post, and this time, we included case control studies and cohort studies instead of only RCTs to expand the sample size. In addition, there is no research on the impact of different types of NSBB on the incidence of the disease, so we performed a meta-analysis to further evaluate and clarify the association between the use of NSBBs (propranolol, nadolol, timolol, and carvedilol) and HCC incidence in patients with cirrhosis.

## Methods

### Search strategy

We searched the PubMed, Web of Science, EMBASE, and Cochrane Library for studies on NSBBs and HCC risk in patients with liver cirrhosis that were published until March 2023. The search used a combination of MESH terms “adrenergic beta-antagonists” and “liver cirrhosis” and free words “nadolol,” “propranolol,” “carvedilol,” “beta-blocker,” and “hepatic cirrhosis” to trace the references of the included literature to supplement the acquisition of relevant literature.

### Inclusion and exclusion criteria

Inclusion criteria: 1) Study type: Cohort study, case control study, or RCT. 2) Patients: Patients with a definite diagnosis of cirrhosis were included. 3) Intervention: Experimental group: only NSBB or NSBB combined with any other treatments; and control group: any intervention except NSBB. 4) Outcome: The HCC incidence or HCC-related mortality was reported in the study outcome.

Exclusion criteria: 1) Studies with inaccurate data extraction or missing data. 2) Review articles, letters, comments, and case reports. 3) If data were duplicated in more than one study, the most recent or informative study was used. 4) The causes of cirrhosis were not comparable between groups.

### Data extraction and quality assessment

Two researchers independently screened the literature, extracted the data, and cross-checked the data. If there was any deviation, the study was discussed or a third researcher assisted in judgment. All relevant texts, tables, and figures were reviewed for data extraction. For the literature lacking data, the original authors were contacted to supplement the literature. The extracted data included the following: 1) basic information of the included studies: the author’s name, publication year, and country; 2) basic characteristics of the subjects: sample size, age, sex, Child-Pugh score, and etiology of cirrhosis; 3) intervention group: type, dose, and course of treatment of NSBB; 4) control group: details and course of intervention; 5) key elements of bias risk assessment; and 6) main data of the outcome and follow-up time. The included studies were evaluated for RCTs by the Cochrane handbook and for cohort and case‒control studies by the Newcastle‒Ottawa Quality Assessment Scale. The GRADEpro tool was also used to calculate the certainty of the evidence.

### Statistical analysis

Stata 17 was used for meta-analysis. As the HCC incidence in the general population is relatively low, the relative risks (RRs) and hazard ratios (HRs) were considered as an approximation of ORs. The I^2^ statistic was used to evaluate the included studies. Between 0 and 25%, heterogeneity was considered insignificant; 25–50%, moderate heterogeneity; 50–75%, substantial heterogeneity; and 75–100%, large heterogeneity. If I^2^ < 25%, the fixed-effects model was used for meta-analysis. If I^2^ ≥ 25%, there was statistical heterogeneity among the study results, and the source of heterogeneity needed to be further analyzed. After excluding obvious clinical and methodological heterogeneity, a random-effects model was preferred.

Subgroup analysis was used to analyze the causes of heterogeneity. Sensitivity analysis was performed by reconducting the meta-analysis after sequentially excluding individual studies and evaluating the differences between the results after exclusion and the original pooled results. Publication bias was visually judged by drawing funnel plots and quantitatively evaluated by Egger’s test and Begg’s test (*p* < 0.05 was taken to indicate significant publication bias). The test level was *α* = 0.05.

## Results

### Study characteristics

**FIGURE 1:**
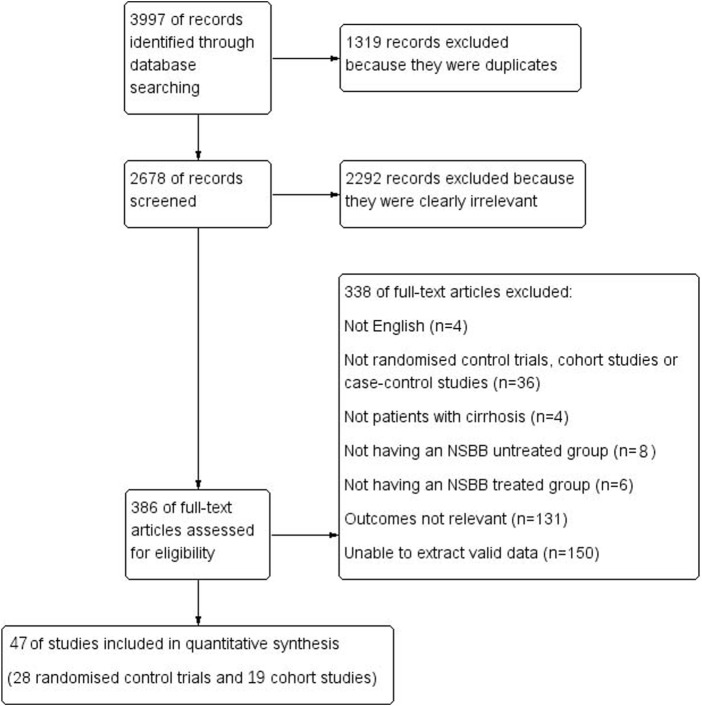
Flow diagram.

From 3,997 studies, we finally selected 47 studies, including 19 cohort studies and 28 RCTs. A total of 1,319 replicates were excluded; 2,292 studies that clearly did not meet the requirements after reading the title and abstract were excluded; 131 articles were excluded because the number of people with HCC or who died from HCC was not reported; 150 studies were excluded because the full text was not available or valid data could not be extracted; 36 articles were excluded because they were not the required type of study (conference abstracts, reviews, and meta-analyses); 4 studies did not assess patients with cirrhosis; and 14 studies did not have an NSBB-untreated group/NSBB-treated group (Figure 1).

The proportion of males varied from 51.4 to 83.1%, and the mean age ranged from 46 to 68 years. Viral hepatitis and alcoholic liver diseases were the leading hepatological causes of cirrhosis in the enrolled patients. In the experimental group, propranolol (*n* = 30), nadolol (*n* = 14), timolol (*n* = 1), and carvedilol (*n* = 9) were used. NSBBs were used as monotherapy (*n* = 34), combined with EVL (*n* = 7), ivabradine (*n* = 1), EIS (*n* = 1), and ISMN (*n* = 5). In the control group, there were placebo (*n* = 8), TIPS (*n* = 4), band ligation (*n* = 15), and not reported (*n* = 14).

We were able to extract data on the incidence of HCC in 38 cases, the mortality of HCC in 26 cases, and the all-cause mortality in 39 cases from 47 trials. Kang2021 patients were divided into primary prophylaxis and secondary prophylaxis groups according to prophylactic treatment of esophageal varices, Wijarnpreecha2021 patients were divided into three groups, namely, propranolol, nadolol, and carvedilol, according to the types of NSBB, and all can be extracted data. In the analysis of the relationship between HCC incidence and NSBB, 17 were RCTs and 21 cohort studies were performed. In the analysis of HCC-related mortality, 26 studies were able to extract relevant data, including six cohort studies and 20 RCTs. In the analysis of all-cause mortality, 39 studies were able to extract relevant data, including 12 cohort studies and 27 RCTs (Table 1). The observational studies were evaluated by the Newcastle–Ottawa Scale, and the RCTs were evaluated by the Cochrane Handbook. The included studies were of high quality after evaluation and scoring, which indicated that the two groups of patients were comparable at baseline (Table 2) (Table 3).

**TABLE 1:** Characteristics of included studies.

Study (year)	Design	Region	Intervention	Control	Number of patients	Duration of NSBB (mean, months)	Dose of NSBB	Etiology of cirrhosis	Complication
Alexandrino et al. (1988)	RCT	Europe	Propranolol	ES	65	28.2	Mean: 130+-65 mg/day (range 40–360)	Alcoholic: 26; non-alcoholic: 6	\
								Nodular regenerative hyperplasia: 1	
								Idiopathic portal thrombosis: 1	
Angelico et al. (1993)	RCT	Europe	Propranolol	ISMN	118	29.2	Median: 60 mg/day (range 20–120)	Alcoholic: 24.6%	Ascites: 38.6%
									Varices: 61
Borroni et al. (2002)	RCT	Europe	Nadolol	ISMN	52	1.3	Mean: 68+-7 mg/day (range 40–120)	Viral: 13	\
								Alcoholic: 5	
								Viral + alcoholic: 6	
								Others: 1	
Chen et al. (2022)	Cohort	Asia	Propranolol	37 statins others NR	3,576	34.3	\	HBV: 561	Variceal bleeding: 169
								HCV: 366	
								Alcoholic: 518	
								Hypertension: 530	
								Cerebrovascular accident: 120	
								Acute coronary syndrome: 146	
								Myocardial infarction: 9	
								Peripheral vascular disease: 42	
								Dementia: 8	
								Dyslipidemia: 158	
								Diabetes mellitus: 514	
								Peptic ulcer disease: 1,141	
								Chronic kidney disease: 173	
Cholongitas et al. (2006)	Cohort	Europe	Propranolol	\	134	24.0	\	HBV: 14	None: 13
								HCV: 6	Gastrointestinal bleeding: 10
								Alcoholic: 3 PBC/PSC: 1	Encephalopathy: 2
								Unknown: 9	
Conn et al. (1991)	RCT	Europe	Propranolol	Placebo	102	17.1	Median: 80 mg/day	Alcoholic: 39	Porto-systemic encephalopathy: 6
							Mean: 132 mg/day		Ascites: 22
									Varices: 51
De La Peña et al. (2005)	RCT	Europe	Nadolol + EVL	Band ligation	80	17.5	Mean: 58 mg/day (range 10–120)	Alcoholic: 27	Previous history of variceal bleeding: 2
								Viral: 12	
								Others: 4	
Escorsell et al. (2002)	RCT	Europe	Propranolol + ISMN	TIPS	91	15.4	\	Alcoholic: 25	Previous ascites: 24
								Others: 19	Previous encephalopathy: 7
Giannelli et al. (2020)	Cohort	Europe	Propranolol	\	584	\	\	HCV: 76	Refractory ascites: 99
								HBV: 35	
								Alcoholic: 141	
								Biliary diseases: 22	
								Others: 17	
Gimenez et al. (2018)	Cohort	Europe	Propranolol(28)	\	63	19.9	28P, range 20–120 mg/day	Alcoholic: 15	Encephalopathy: 25
			Nadolol(2)				2N, range 40 mg/day	Viral: 11	Upper digestive bleeding: 18
								Others: 4	Ascites: 29
Gonzalez-Suarez et al. (2006)	Cohort	Europe	Nadolol + ISMN	ES	230	25.6	\	Alcoholic: 59	Ascites: 70
				Band ligation				HCV: 43 Others: 13	Encephalopathy: 20
									Portal thrombosis: 5
									Previous SBP: 5
Groszmann et al. (2005)	RCT	Europe	Timolol	Placebo	213	54.6	Median: 10.8 mg/day (range 1.25–80.0)	Alcoholic: 26	\
								HCV: 67	
								HBV: 6	
								Cryptogenic: 5	
								Others: 4	
Ideo et al. (1987)	RCT	Europe	Nadolol	Ranitidine placebo	79	24.4	Range 40–120 mg/day	Alcoholic: 14	Treatable ascites: 10
									Encephalopathy: 2
Kang et al. (2021)	Cohort	Asia	Propranolol ± EVL	\	740	39.1	Median: 120 mg/day (IQR 80-160)	Alcoholic: 403	\
								Viral: 125	
								Others: 21	
								Severe cardiac disease: 0	
								Renal dysfunction: 8	
Kim et al. (2012)	Cohort	Asia	Propranolol	\	273	41.9	Mean 29.6+-11.0 mg/day (range 20–60)	Alcoholic: 49	Presence of esophageal varix: 138
								HBV: 68	Diabetes: 38
								HCV: 12	Hypertension: 9
								Cryptogenic: 9	
Lay et al. (2006)	RCT	Asia	Propranolol	Band ligation	100	34.5	Mean: 68.2+-32.8 mg/day	Alcoholic: 49	Ascites: 9
								HBV: 68	
								HCV: 12	
								Cryptogenic: 9	
Lebrec et al. (1988)	RCT	Europe	Nadolol	Placebo	106	12.0	\	Alcoholic: 39	\
								HBV: 6; primary biliary cirrhosis: 3	
								Cryptogenic: 5	
Lee et al. (2020)	Cohort	Asia	Propranolol	\	1,038	32.4	\	Others: 28	Ascites: 139
								Viral hepatitis: 291	Gastroesophageal varices: 150
								Alcoholic: 200	SBP: 20
Leithead et al. (2014)	Cohort	Europe	Propranolol(78)	\	208	2.9	Median: propranolol 80 mg/day (range 10–240) median: carvedilol 6.25 mg/day (range 3.125–12.5)	Alcoholic: 42	Previous variceal hemorrhage: 29
			Carvedilol(26)					HCV: 24	Refractory ascites: 39
								Cholestatic: 18	Hepatorenal syndrome (type 2): 5
								Non-alcoholic fatty liver disease: 10	
Lo et al. (2000)	RCT	Asia	Nadolol + EVL + sucralfate	Band ligation	122	22.0	Mean: 60+-18 mg/day (range 40–120)	Alcoholic: 17	Ascites: 33
								Post-hepatitis: 41	Encephalopathy: 4 Gastropathy: 15
								Cryptogenic: 2	
Lo et al. (2004)	RCT	Asia	Nadolol	Band ligation	100	22.6	Mean: 60+-20 mg/day (range 20–80)	Alcoholic: 10	Ascites: 20 Gastric varices: 18
								HBV: 19	
								HCV: 13	
								HBV + HCV: 3	
								Cryptogenic: 5	
Lo Iacono et al. (2008)	RCT	Asia	Nadolol + ISMN	Band ligation	121	81.0	Mean: 48+-10 mg/day (range 20–120)	Alcoholic: 22	Ascites: 36
								HBV: 20	Encephalopathy: 2
								HCV: 12	Previous bleeding: 22
								HBV + HCV: 4	
								Cryptogenic: 3	
Luo et al. (2015)	RCT	Asia	Propranolol + EVL	TIPS	73	20.9	Mean: 65.4+-26.7 mg/day	HBV: 24	Hepatic encephalopathy: 3
								HCV: 2	Ascites: 20
								Alcoholic: 4	
								Others: 6	
Lv et al. (2017)	RCT	Asia	Propranolol + EVL	TIPS	49	31.4	Median: 80 mg/day (range 20–170)	HBV: 22	Ascites: 17
								HCV: 0	Previous hepatic encephalopathy: 1
								Alcoholic: 0	Previous bleeding: 12
								Autoimmune hepatitis: 1	
								HBV + autoimmune hepatitis: 1	
								Cryptogenic: 1	
McDowell et al. (2021)	Cohort	Europe	Carvedilol	Band ligation	152	93.6	\	Alcoholic: 77	\
Merkel et al. (2004)	RCT	Europe	Nadolol	Placebo	161	36.0	Mean: 62+-25 mg/day (range 40–160)	Alcoholic: 47; Viral: 34	Ascites: 18
								Others: 2	
Metwally et al. (2022)	RCT	Africa	Cyanoacrylate injection + EVL + carvedilol(24)	Band ligation	74	8.6	\	HCV: 41	Diabetes: 7
			EVL + carvedilol(25)						Gastrointestinal bleeding: 12
Ngwa et al. (2016)	Cohort	America	Propranolol(36)	\	170	\	\	HCV: 27	Spontaneous bacterial peritonitis: 2
			Carvedilol(10)					Alcoholic: 23	Hepatorenal syndrome: 2
			Nadolol(19)					NASH: 20	Hepatopulmonary syndrome: 0
									Porto-pulmonary hypertension: 2
Nkontchou et al. (2012)	Cohort	Europe	Propranolol	\	291	56.1	160 mg/day or 40 mg twice a day	Mixed etiology: 15	Diabetes: 17
Pascal and Cales (1989)	RCT	Europe	Propranolol	Placebo	228	14.5	Mean: 162.4+-85.3 mg/day	\	\
Patch et al. (2002)	RCT	Europe	Propranolol	Band ligation	102	10.9	Median: 80 mg/day (range 40–240)	Alcoholic: 32	\
Premkumar et al. (2020)	RCT	Asia	Carvedilol ± ivabradine	SMT	189	17.9	Median: 9.37 mg/day (range 3.125–12.5)	\	\
Psilopoulos et al. (2005)	RCT	Europe	Propranolol	Band ligation	60	27.9	Mean: 60.3+-13.3 mg/day	HBV: 12	\
								HCV: 5	
								HBV + HCV: 2	
								Alcoholic: 7	
								Cryptogenic: 2	
								Primary biliary cirrhosis: 1	
								Autoimmune: 1	
Sauer et al. (1997)	RCT	Europe	Propranolol + EIS	TIPS	83	17.4	\	Alcoholic: 26	\
								Viral: 10	
								Others: 5	
Scheiner et al. (2017)	Cohort	Oceania	Propranolol and carvedilol	\	176	33.9	Mean: P,95.6 ± 31.2 mg/day mean: C,19.2 ± 11.0 mg/day	Alcoholic: 47	Ascites: 31
								Viral: 25	
								Mixed: 5	
								Others: 8	
								Unknown: 8	
Singh et al. (2022)	RCT	Asia	Propranolol	Band ligation	160	9.6	\	Alcoholic: 38	Spontaneous bacterial peritonitis: 19
								Non-alcoholic steatohepatitis: 11	Hepatic encephalopathy: 15
								HCV: 11	Acute kidney injury: 13
								HBV: 5	
								HCV and alcohol: 4	
								HBV and alcohol: 2	
								Cryptogenic: 5	
								Autoimmune related: 4	
								Budd Chiari syndrome: 0	
Sinha et al. (2017)	Cohort	Europe	Carvedilol	Band ligation	264	2.4	Median: 12.5 mg (range 6.25–12.5)	ALD: 94	Previous variceal bleed: 42
								NAFLD: 15	
								Viral: 5	
								Others: 12	
Snoga et al. (2020)	Cohort	America	Propranolol and nadolol	\	255	\	Median: P, 20 mg/day (range 20–40)	Alcoholic: 156	Renal replacement therapy: 1
							Median: N, 30 mg/day (range 20–100)	HCV: 116	Endoscopic variceal ligation: 214
									Hepatic encephalopathy: 113
									Ascites: 138
									Previous variceal bleed: 50
Tripathi et al. (2009)	RCT	Europe	Carvedilol	Band ligation	152	26.2	12.5 md/day	Alcoholic: 57	Ascites: 49%
								Abstained: 17	
Villanueva et al. (1996)	RCT	Europe	Nadolol + ISMN	ES	86	18.4	Mean: 110+-70 mg/day	Alcoholic: 25	Previous bleeding: 5
								Viral: 13	Ascites: 17
									Encephalopathy: 8
Villanueva et al. (2001)	RCT	Europe	Nadolol + ISMN	Band ligation	144	21.8	Mean: 96+-56 mg/day	Alcoholic: 33	Previous bleeding: 10
								Viral: 24	Associated diseases: 28
								Alcohol + virus: 10	Ascites: 47
									Encephalopathy: 11
Villanueva et al. (2019)	RCT	Europe	Propranolol(67)	Placebo	201	36.9	P: median: 80 mg/day (IQR 40-120) mean: 95 mg/day	Alcoholic: 19	Diabetes: 22
			Carvedilol(33)				C: median: 18.8 mg/day (IQR 12.5-25) mean: 19 mg/day	HCV: 54	Dyslipidaemia: 12
								Alcoholic + HCV: 9	Arterial hypertension: 45
								NASH: 5	
								Others: 13	
Villeneuve et al. (1986)	RCT	America	Propranolol	Placebo	79	22.3	Mean: 103+-7 mg/day	Alcoholic: 31	Previous episodes of bleeding: 14
								Post-hepatitic: 3	
								Cryptogenic: 4	
								Biliary: 3	
								Portal vein thrombosis: 1	
								Idiopathic portal hypertension: 0	
Wallen et al. (2017)	Cohort	Oceania	Propranolol	\	72	\	Range 10–40 mg/day	Alcoholic: 11	Gastroesophageal varices: 25
								Viral: 9	Refractory ascites: 6
								Others: 8	Hepatopulmonary syndrome: 1
									Porto-pulmonary hypertension: 0
Wijarnpreecha et al. (2021)	Cohort	America	Propranolol	\	107428	58.7	\	\	\
			Carvedilol and nadolol						
Yeh et al. (2019)	Cohort	Asia	Propranolol	\	13,792	49.2	\	\	\
Yoo et al. (2020)	Cohort	Asia	Propranolol + EVL	Band ligation	252	\	\	Viral: 70 Non-viral: 102	\

ISMN, isosorbide mononitrate; EVL, endoscopic variceal band ligation; ES, endoscopic treatment with sclerotherapy; SMT, including dietary modifications, diuretics, lactulose, and/or rifaximin in patients with prior encephalopathy; TIPS, transjugular intrahepatic portosystemic shunt; SBP, spontaneous bacterial peritonitis; CAD, coronary artery disease; NASH, non-alcoholic steatohepatitis.

**TABLE 2:** Bias assessment.

	Allocation sequence generation	Allocation sequence concealment	Blinding of participants	Blinding of outcome assessors	Incomplete outcome data	Selective outcome reporting	Other bias
Alexandrino et al. (1988)	Low	Unclear	High	Unclear	Unclear	Unclear	Unclear
Angelico et al. (1993)	Unclear	Unclear	Unclear	Unclear	Unclear	Unclear	Low
Borroni et al. (2002)	Unclear	Low	High	High	Unclear	Unclear	Unclear
Conn et al. (1991)	Low	Unclear	High	Low	Unclear	Unclear	Low
DeLaPeña et al. (2005)	Low	Low	Low	High	Unclear	Unclear	Unclear
Escorsell et al. (2002)	Low	Low	High	High	Unclear	Unclear	Low
Groszmann et al. (2005)	Low	Low	Low	Low	Unclear	Unclear	Low
Ideo et al. (1987)	Low	Unclear	Low	High	Unclear	Unclear	Low
Lay et al. (2006)	Unclear	Unclear	High	High	Unclear	Unclear	Unclear
Lebrec et al. (1988)	Low	Unclear	Unclear	High	Unclear	Unclear	Unclear
Lo et al. (2000)	Low	Low	High	High	Unclear	Unclear	Low
Lo et al. (2004)	Low	Low	High	High	Unclear	Unclear	Low
Lo Iacono et al. (2008)	Low	Low	High	High	Unclear	Unclear	Low
Luo et al. (2015)	Low	Unclear	High	Unclear	Low	Unclear	Low
Lv et al. (2017)	Low	Low	High	Low	Low	Unclear	Low
Merkel et al. (2004)	Low	Low	Low	High	Unclear	Unclear	Low
Metwally et al. (2022)	Low	Low	Low	Low	Low	Unclear	Low
Pascal and Cales. (1989)	Low	Low	Unclear	High	Unclear	Unclear	Unclear
Patch et al. (2002)	Low	Low	High	High	Unclear	Unclear	Unclear
Premkumar et al. (2020)	Unclear	High	High	High	Unclear	Unclear	Unclear
Psilopoulos et al. (2005)	Low	Unclear	High	High	Unclear	Unclear	Unclear
Sauer et al. (1997)	Low	Low	High	High	Unclear	Unclear	Low
Singh et al. (2022)	Low	Low	High	High	Unclear	Unclear	Low
Tripathi et al. (2009)	Unclear	Low	High	High	Unclear	Unclear	Low
Villanueva et al. (1996)	Low	Low	High	High	Unclear	Unclear	Low
Villanueva et al. (2001)	Low	Low	High	High	Unclear	Unclear	Low
Villanueva et al. (2019)	Low	Low	Low	Low	Low	Unclear	Unclear
Villeneuve et al. (1986)	Unclear	Unclear	High	High	Unclear	Unclear	Unclear

**TABLE 3:** The NOS for cohort studies.

	Representativeness of the exposed cohort	Selection of the non-exposed cohort	Ascertainment of exposure	Demonstration that the outcome of interest was not present at the start of the study	Control for important factors^a^	Assessment of the outcome	Follow-up long enough for outcomes to occur	Adequacy of follow-up of cohorts	Total scores
Chen et al. (2022)	1	1	1	1	1	1	1	1	8
Cholongitas et al. (2006)	1	1	1		2	1	1	1	8
Giannelli et al. (2020)	1	1	1			1		1	5
Gimenez et al. (2018)	1	1	1	1	1	1	1	1	8
Gonzalez-Suarez et al. (2006)	1	1	1	1		1	1	1	7
Kang et al. (2021)	1	1	1	1	1	1	1	1	8
Kim et al. (2012)	1	1	1	1	2	1	1	1	9
Lee et al. (2020)	1	1	1		2	1	1	1	8
Leithead et al. (2014)	1	1	1		2	1		1	7
McDowell et al. (2021)	1	1	1	1		1	1	1	7
Ngwa et al. (2016)	1	1	1		2	1		1	7
Nkontchou et al. (2012)	1	1	1	1	1	1	1	1	8
Scheiner et al. (2017)	1	1	1	1	2	1	1	1	9
Sinha et al. (2017)	1	1	1		2	1	1	1	8
Snoga et al. (2020)	1	1	1		1	1		1	6
Wallen et al. (2017)	1	1	1		2	1		1	7
Wijarnpreecha et al. (2021)	1	1	1		2	1	1	1	8
Yeh et al. (2019)	1	1	1	1		1		1	6
Yoo et al. (2020)	1	1	1	1	2	1		1	8

^a^ A maximum of two stars could be awarded for this item. Studies that are controlled for age and sex received one star, whereas studies that are controlled for additional covariates received another star.

### Incidence of HCC

**FIGURE 2:**
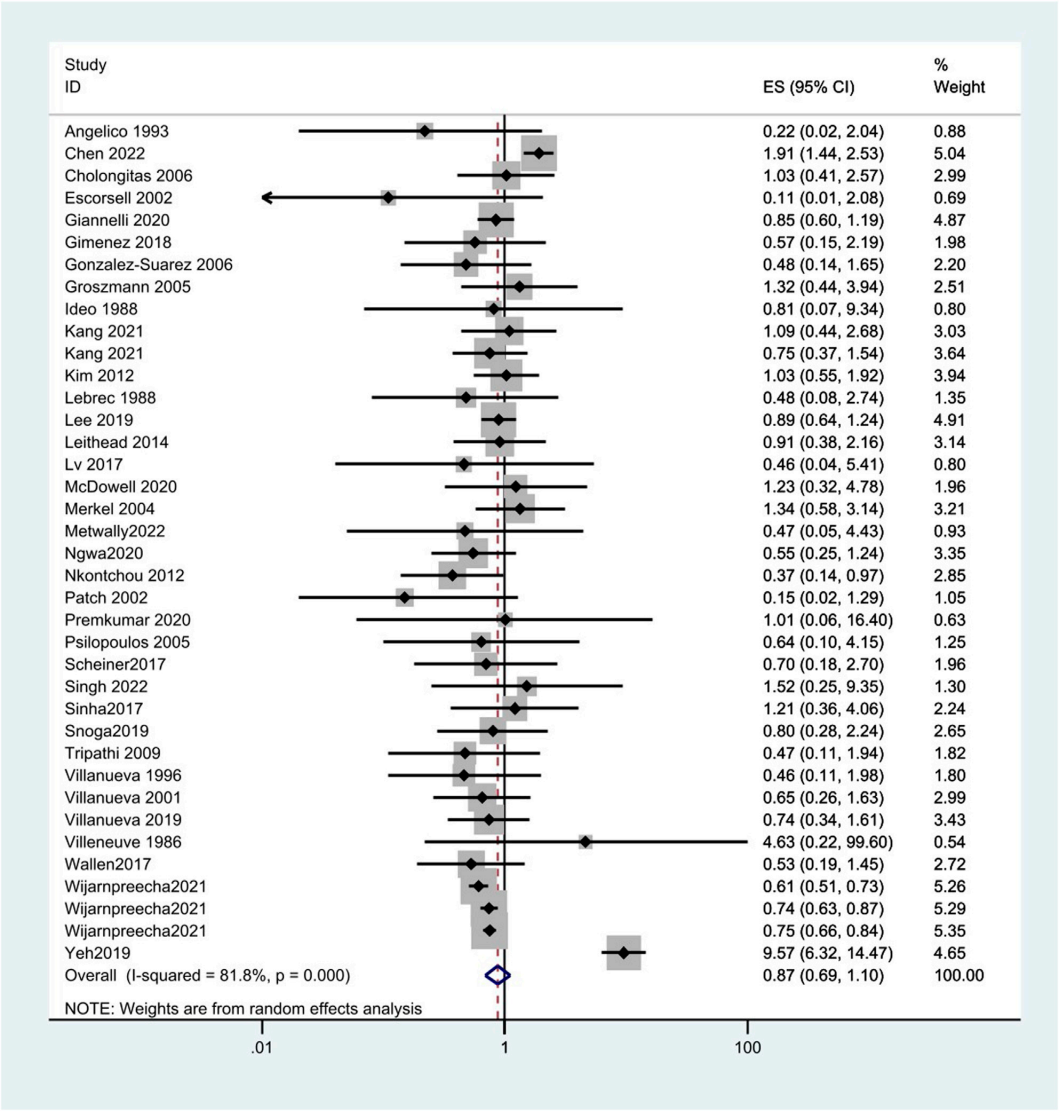
Forest plots of hepatocellular carcinoma incidence associated with NSBBs and random-effects meta-analysis.

**FIGURE 3:**
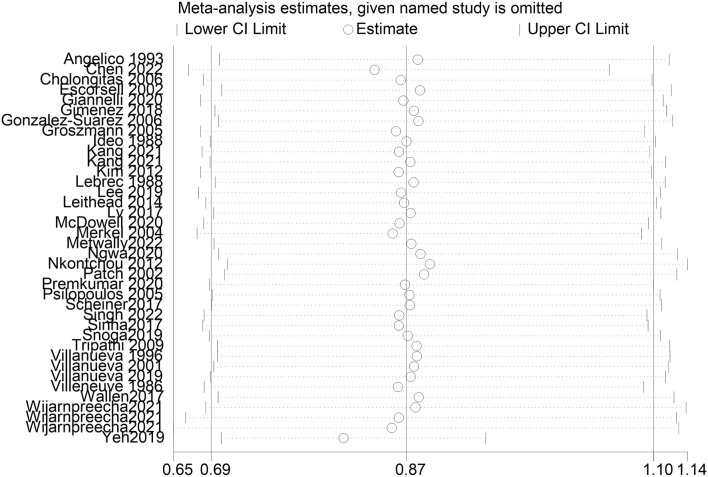
Sensitivity analysis.

A total of 38 studies included 410 of 8,450 cirrhotic patients in the NSBB group and 432 of 8,311 cirrhotic patients in the control group. With OR as the effect index, the fixed-effects model showed OR = 0.84, 95%CI 0.78–0.9, *p* = 0.000, and I^2^ = 81.8%, which was considered to indicate large heterogeneity. The random-effect model was adjusted to show that NSBBs could reduce the incidence of HCC (OR = 0.87 and 95%CI 0.69–1.10) (Figure 2). Egger’s test: *p* = 0.786 and Begg’s test: *p* = 0.744 suggested that there is no publication bias. Sensitivity analysis showed that the overall risk estimates were not substantially modified by any single study (Figure 3).

The results confirmed that NSBBs can reduce the HCC incidence in subgroup analyses when patients come from Europe (OR = 0.77, 95%CI 0.62–0.96, *p* = 0.765, and I^2^ = 0.0%) and America (OR = 0.71, 95%CI 0.64–0.78, *p* = 0.339, and I^2^ = 11.9%) (Table 4).

**TABLE 4:** Subgroup analysis, random.

	No. of studies	OR(95%CI)	p	I2 (%)
*Incidence of HCC*				
All studies	38	0.87(0.69–1.10)	0.000	81.8
Types				
Propranolol	18	0.94(0.62–1.44)	0.000	89.6
Nadolol	7	0.74(0.64–0.86)	0.796	0.0
Timolol	1	1.32(0.44–3.95)		
Carvedilol	6	0.62(0.52–0.74)	0.776	0.0
Undetailed report	6	0.69(0.48–1.08)	0.938	0.0
Study design				
RCT	17	0.74(0.52–1.04)	0.763	0.0
Cohort	21	0.95(0.71–1.26)	0.000	89.5
Region				
European	20	0.77(0.62–0.96)	0.765	0.0
Asia	9	1.45(0.72–2.93)	0.000	91.3
Africa	1	0.47(0.05–4.42)		
America	6	0.71(0.64–0.78)	0.339	11.9
Oceania	2	0.59(0.26–1.32)	0.747	0.0
*HCC-related mortality*				
All studies	26	1.13(0.80–1.60)	0.998	0.0
Study design				
Cohort	6	1.44(0.80–2.59)	0.921	0.0
RCT	20	0.98(0.64–1.53)	0.995	0.0
Region				
European	17	1.18(0.78–1.78)	0.972	0.0
Asia	8	0.97(0.49–1.90)	0.965	0.0
America	1	2.71(0.11–68.59)		
*Overall mortality*				
All studies	39	0.85(0.69–1.05)	0.000	77.4
Study design				
Cohort	12	1.01(0.68–1.49)	0.000	91.9
RCT	27	0.75(0.63–0.89)	0.614	0.0
Region				
European	22	0.86(0.66–1.12)	0.002	53.7
Asia	13	0.78(0.54–1.12)	0.000	89.3
Africa	1	4.17(0.36–48.44)		
America	2	1.31(0.79–2.19)	0.931	0.0
Oceania	1	0.52(0.25–1.11)		

**FIGURE 4:**
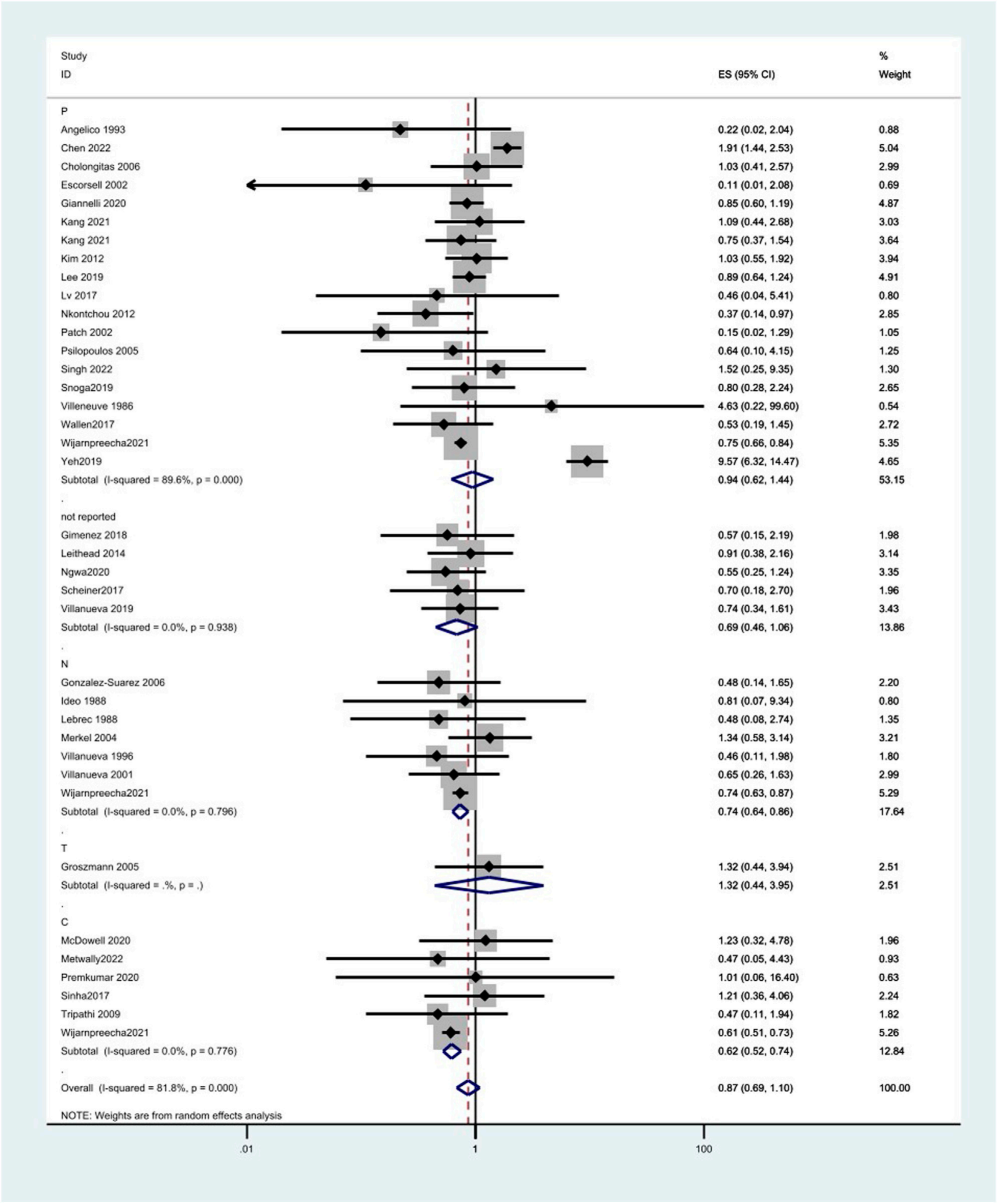
Forest plots of hepatocellular carcinoma incidence associated with different kinds of NSBB and random-effects meta-analysis.

Different types of NSBB have different effects on the incidence of patients with cirrhosis of the liver. There was no significant association between propranolol (OR = 0.94, 95%CI 0.62–1.44, *p* = 0.000, and I^2^ = 89.6%) or timolol (OR = 1.32 and 95%CI 0.44–3.95) and HCC incidence, while the risk of HCC decreases by 26% and 38% with nadolol (OR = 0.74, 95%CI 0.64–0.86, *p* = 0.796, and I^2^ = 0.0%) and carvedilol (OR = 0.62, 95%CI 0.52–0.74, *p* = 0.776, and I^2^ = 0.0%), respectively. Also, the effect of NSBB may vary in ethnicity. Propranolol can reduce HCC incidence in Europe (OR = 0.60, 95%CI 0.36–0.99, *p* = 0.211, and I^2^ = 28.5%) and America (OR = 0.75, 95%CI 0.67–0.85, *p* = 0.503, and I^2^ = 0.0%) (Figure 4) (Table 5).

**TABLE 5:** Subgroup analysis in different NSBBs, random.

Subgroup	No. of studies	OR(95%CI)	p	I^2^ (%)
*Propranolol*	19	0.94(0.62–1.44)	0	89.6
Design				
RCT	7	0.49(0.20–1.19)	0.364	8.5
Cohort	12	1.08(0.68–1.72)	0	93.3
Region				
Europe	7	0.60(0.36–0.99)	0.211	28.5
Asia	8	1.47(0.71–3.05)	0	92.3
America	3	0.75(0.67–0.85)	0.503	0.0
Oceania	1	0.53(0.19–1.46)		
*Nadolol*	7	0.74(0.64–0.86)	0.796	0.0
Design				
RCT	5	0.81(0.48–1.38)	0.643	0.0
Cohort	2	0.73(0.63–0.86)	0.495	0.0
Region				
Europe	6	0.75(0.46–1.22)	0.685	0.0
America	1	0.74(0.64–0.86)		
*Carvedilol*	6	0.62(0.52–0.74)	0.776	0.0
Design				
RCT	3	0.53(0.17–1.61)	0.776	0.0
Cohort	3	0.66(0.48–0.89)	0.337	8.1
Region				
Europe	3	0.93(0.43–2.00)	0.545	0.0
Asia	1	1.01(0.06–16.70)		
America	1	0.61(0.51–0.73)		
Africa	1	0.47(0.05–4.42)		
*Timolol*	1	1.32(0.44–3.95)		

### Overall mortality

A total of 39 trials were 2,732/11,538 in the NSBB and 2,683/11,390 in the control. Taking OR as the effect index, the fixed-effects model showed that OR = 0.96, 95%CI 0.89–1.04, *p* = 0.000, and I^2^ = 77.4% because it was more than 75%, so it was considered to have a large heterogeneity. Therefore, the random-effects model was adjusted to show that the use of NSBBs was not associated with a reduction in all-cause mortality (OR = 0.85 and 95%CI 0.69–1.05). Both Egger’s test *p* = 0.452 and Begg’s test *p* = 0.070 had symmetric good plots and lacked any indication of publication bias.

The results from the random-effects model were confirmed in all subgroup analyses except for the study design. In RCTs, we found a significant difference, with OR = 0.75, 95%CI 0.63–0.89, *p* = 0.614, and I^2^ = 0.0%, suggesting that the use of NSBBs may reduce all-cause mortality in patients with cirrhosis (Table 4).

### HCC-related mortality

A total of 75 of 2,088 patients in the NSBB group and 57 of 1,696 patients in the control group died of HCC. There was no effect of NSBBs on HCC mortality in the fixed-effects model, OR = 1.13, 95%CI 0.80–1.60, *p* = 0.998, and I^2^ = 0.0%, or random-effects model, OR = 1.09 and 95%CI 0.76–1.57. Because Begg’s and Egger’s tests have the same statistical problem, but the test efficiency of Begg is lower than that of Egger, Egger’s test result should prevail for analysis. So, there are no small study effects (Egger’s test *p* = 0.180 and Begg’s test *p* = 0.050). The result was confirmed in the subgroup and sensitivity analyses (Table 4).

## Discussion

In this meta-analysis, we examined RCTs and cohort studies on the relationship between NSBB use and HCC. We found that, in comparison with non-use, nadolol and carvedilol may reduce the HCC incidence in patients with cirrhosis, but no significant association was found in propranolol and timolol. Also, the effect of NSBB may vary in ethnicity. Propranolol can reduce HCC incidence in Europe and America. There was no significant association with HCC-related mortality or all-cause mortality.

We found that nadolol and carvedilol can decrease the risk of HCC incidence by 26 and 38%, respectively, and propranolol can reduce the risk of HCC incidence by 40 and 25% in Europe and America, respectively. The depressed risk of HCC in cirrhotic patients treated with NSBBs in our meta-analysis is consistent with the results reported in previous studies. In a meta-analysis by Thiele et al., NSBBs had the potential to decrease the incidence of HCC with a risk difference of 2.6% (Thiele et al., 2015).

HCC is the third leading cause of cancer death worldwide, with a relative 5-year survival rate of approximately 18%. Morbidity and mortality were similar, highlighting the poor prognosis associated with the disease (Siegel et al., 2022). Cirrhosis is considered to be a major cause of HCC, and the incidence is increasing (Flemming et al., 2014). For these patients, liver fibrosis limits cancer treatment. Therefore, it is necessary to prevent HCC in patients with cirrhosis. The mechanism by which NSBBs reduce the incidence of HCC may be that NSBBs affect not only the portal pressure but also non-hemodynamic parameters. NSBBs can induce apoptosis and S-phase arrest in human HCC cell lines by inhibiting nuclear factor-κb signaling and block camp-dependent intracellular signaling and camp-dependent release of epidermal growth factor and PKA-dependent release of vascular endothelial growth factor, thereby reducing HCC cell invasion and migration (Al-Wadei et al., 2009; Liao et al., 2010). Even reducing angiogenesis to block the nutrient supply to the tumor may prevent its proliferation and cytotoxicity to HCC cells. In addition, by promoting intestinal transport, NSBBs could promote the homeostasis of gut microbiota and reduce its translocation, thereby reducing the proinflammatory cytokine load in the liver caused by gut microbiota metabolites (Powe et al., 2010; Chim et al., 2012; Thiele et al., 2015). Currently, NSBB applied in the clinic can be divided into two categories. The first type acts on the β1 and β2 receptors of the heart and peripheral blood ducts at the same time, among which the most commonly used drugs include propranolol, nadolol, and timolol. The second type is a novel non-selective β-blocker, which has a weak anti-α adrenergic effect and can dilate peripheral blood ducts. The representative drugs are carvedilol and nipradilol. Among them, carvedilol has additional effects on calcium influx and ROS-mediated inhibition of PI3K/AKT signaling, both of which have inhibitory effects on carcinogenesis or tumor progression (Snoga et al., 2020).

The results showed that there is no significant association between NSBBs and the incidence of HCC in patients with cirrhosis, but there was large heterogeneity among the included studies. Therefore, we conducted a subgroup analysis according to the NSBB type and region. There was no significant association between propranolol (OR = 0.94, 95%CI 0.62–1.44, and I^2^ = 89.6%) or timolol (OR = 1.32 and 95%CI 0.44–3.95) and HCC incidence, while the risk of HCC decreases with nadolol (OR = 0.74 and 95%CI 0.64–0.86) and carvedilol (OR = 0.62 and 95%CI 0.52–0.74). Carvedilol has anti-α-1 receptor activity and can add non-selective β-blocking activity. This additional effect of carvedilol improves its pharmacodynamic effects, especially hypotensive effects, compared with conventional NSBBs. Carvedilol was, therefore, associated with lower rates of rebleeding, liver-related mortality, and further non-hemorrhagic decompensation (Sinha, 2022). Nadolol is a synthetic NSBB. Unlike propranolol, nadolol is not metabolized by the liver and is excreted mainly by the kidneys and to a lesser extent in the feces, thus reducing the burden on the liver in patients with cirrhosis. Nadolol has no intrinsic sympathomimetic activity, and the receptor does not produce any agonizing effect, has little myocardial depressor activity compared with propranolol, and does not have an anesthesia-like membrane stabilizing effect (Dreyfuss et al., 1977). In addition, nadolol, which is less lipophilic and, therefore, does not cross the blood–brain barrier, may reduce the risk of CNS adverse events, such as sleep disturbances, behavioral changes, and effects on memory (McAinsh and Cruickshank, 1990). In the analysis of the efficacy of propranolol, there was substantial heterogeneity. Because of racial differences in the sensitivity of the NSBBs, we further compared the efficacy of propranolol in patients from different regions. We found a significant preventive effect in Europe and America but not in Asia. Zhou said Asian populations require lower doses of propranolol to achieve target blood pressure and heart rate than Caucasians and, therefore, use lower doses per day than Caucasians (Zhou et al., 1989). One study showed that low-dose NSBBs had no effect on the overall survival and hepatocellular carcinoma-free survival in patients with cirrhosis (Kim et al., 2012). Thus, the benefit of propranolol was greater in white populations than in Asian populations. In only one of the studies we included did patients consume timolol, so the conclusions drawn lack credibility.

Our findings suggest that NSBB use is not associated with all-cause mortality in patients with cirrhosis. This result was supported by Snoga et al.’s study, with similar mortality at 24 months in the NSBB and non-NSBB groups (32.0% *vs.* 38.5% and *p* = 0.51) in a dual-center study (Snoga et al., 2020). There are some data showing that patients with end-stage cirrhosis have spontaneous bacterial peritonitis (Mandorfer et al., 2014), patients receiving higher doses of refractory ascites (Sersté et al., 2010) or patients with more severe circulatory dysfunction (Sersté et al., 2011) and NSBBs may be harmful. However, some studies have reached completely opposite conclusions. Bernard et al. showed that NSBB treatment significantly reduced the rebleeding rate of varicose veins and improved the 2-year survival rate in his meta-analysis (Bernard et al., 1997). Madsen et al. 23 found an association between NSBB dose and mortality in patients with spontaneous bacterial peritonitis. Treatment with low-dose NSBB (80 mg) was associated with a reduced risk of death, whereas treatment with high-dose NSBB (160 mg) tended to increase the risk of death (Madsen et al., 2016). The different results of these studies suggest that the effect of NSBB on the mortality of patients with cirrhosis may be affected by many factors, such as the dose used and the patient’s condition. Therefore, the relationship between NSBB and mortality in patients with cirrhosis needs to be further answered by more prospective studies.

There was no significant relationship between NSBB use and HCC-related mortality in our study. However, in a cohort of HCC patients, NSBBs were associated with lower liver cancer mortality in patients with primary HCC (Marsdottir et al., 2020). At the same time, a meta-analysis in 2022 also showed that beta-blocker use could be related to the prolonged survival of patients with HCC (Chang and Lee, 2022). A meta-analysis in 2015 explained that the average effect of β-blocker use after diagnosis but not before diagnosis is beneficial for the survival of cancer patients (Zhong et al., 2016). Because the primary outcome in our study was not HCC-related mortality, the collected data in the included studies were limited and insufficient to draw firm conclusions. Future well-designed prospective RCTs are needed to determine the full potential impact of NSBB use on HCC-related mortality.

Our meta-analysis has several strengths. First, we took 19 cohort studies and 28 RCTs into consideration, including a large sample size. Second, we retrieved many studies, and we enhanced our statistical power for examining the association. Third, our studies are not affected by publication bias, so the probability of publishing a study does not depend on the strength or direction of the associations. Fourth, we further explored the effect of different types of NSBB on the incidence of HCC in patients.

Here, we still have some limitations. First, we included only published surveys, but some relevant unpublished data were not included, which may affect our results. Second, some studies reported in this meta-analysis utilized a retrospective cohort design, which is more susceptible to recall and selection biases than a prospective study or RCTs. Third, heterogeneity may be introduced through the methodological differences among the studies, including different diagnostic criteria. Fourth, data regarding the dose of the NSBB used were not consistently provided. It was difficult to evaluate whether certain types and specific doses were more influential. Fifth, because the researchers included in the literature failed to provide specific information on the incidence of HCC in patients with various causes of cirrhosis, we were unable to conduct a subgroup analysis on the impact of different causes of cirrhosis on the outcome of the main study. It is difficult to assess whether certain types of etiology, such as HBV infection, have a particular effect on the use of NSBB and the incidence of HCC. Furthermore, while our funnel plot did not suggest significant publication bias, our analyses did demonstrate moderate heterogeneity across studies, which further contributed to the overly low certainty of the evidence.

Cirrhosis is the result of chronic liver diseases of any cause caused by progressive liver damage and liver fibrosis. Consequently, cirrhosis leads to portal hypertension and liver dysfunction, which in turn develop into multiple adverse complications, resulting in impaired quality of life, loss of social and economic productivity, and reduced survival (Premkumar and Anand, 2022). Cirrhosis is associated with clinically significant portal hypertension due to structural and dynamic changes in the liver and systemic circulation, including the activation of several fibrotic and inflammatory pathways. The Baveno VI Consensus recommends that patients with cirrhosis should be screened for esophageal varices to avoid bleeding and death. High-risk patients should receive conventional NSBBs (propranolol or nadolol), carvedilol, or EVL for the primary prevention of esophageal variceal rupture (de Franchis and Baveno, 2015). At present, these three methods or their combination are still used in clinical treatment. A review comparing three prevention strategies in conjunction with the previous studies showed that EVL may be superior to medical therapy in preventing the first bleeding episode, but the NSBB medical therapy appears to prevent different complications of liver disease and may play a more significant role in reducing mortality (de Mattos Â et al., 2021). This advantage may be related to the fact that NSBBs can act on the pathophysiology of portal hypertension, while EVL only acts on esophageal varices. NSBBs have been proven to reduce the incidence of spontaneous bacterial peritonitis and improve the quality of life of patients with cirrhosis by improving the immune response and reducing the risk of bacterial infection (Praharaj and Anand, 2022). NSBBs induce apoptosis and S-phase arrest in human HCC cell lines. The effect of reducing the invasion and migration of HCC cells can prevent the transformation of liver cirrhosis into hepatocellular carcinoma (Wang et al., 2018; Yeh et al., 2019). In addition, compared with EVL, NSBBs have the advantages of low cost and easy management. To detect the recurrence of varicose veins, EVL requires long-term endoscopic detection, which requires high medical resources (Gimenez et al., 2018). However, a recent clinical trial has shown that the compensatory cardiac response to vasodilation in refractory ascites relies heavily on sympathetic hyperactivation and that β-blockers attenuate sympathetic hyperdrive of the cardiac function, impede cardiac output, reduce renal perfusion below critical thresholds, and impair renal function (Téllez et al., 2020).Therefore, although NSBBs are recommended for clinical treatment, combined EVL therapy can be considered for patients with different conditions to improve the ability to prevent bleeding episodes and reduce the occurrence of related adverse complications.

## Conclusion

Different types of NSBB have different effects on the incidence of patients with cirrhosis of the liver, where nadolol and carvedilol can reduce the risk. Also, the effect of NSBBs may vary in ethnicity. Propranolol can reduce HCC incidence in Europe and America. Our meta-analysis with the published studies did not observe harm or benefit to HCC-related mortality and all-cause mortality associated with NSBBs. In the future, RCTs are needed to ascertain the relationship. There was heterogeneity across studies, and the relationship between NSBBs and HCC in cirrhosis should be further interpreted with well-adjusted data and better-organized clinical trials. In addition, the dose of different types of NSBB on the prevention of HCC should also be further discussed.

## Data Availability

The original contributions presented in the study are included in the article/Supplementary Material. Further inquiries can be directed to the corresponding author.
